# A Brief Sketch of a Few Extraordinary Surgical Cases

**Published:** 1872-02-15

**Authors:** E. R. Willard

**Affiliations:** Wilmington, Illinois


### A BRIEF SKETCH OF A FEW EXTRAORDINARY SURGICAL CASES.

BY E. R. WILLARD, M.D., WILMINGTON, ILLINOIS.

*Continued from page* 588, 1871.

*Case* 5.—Oct. 9th, 1868, I was sent for in great haste to see Mr. J. R., some eight miles distant, who had just been thrown from his buggy, and was reported as lying in an insensible condition, with blood oozing from his mouth, nose, and ears.

Upon a careful survey of the case, after reaching the house, I discovered an extensive fracture of the left temporal and parietal bones, extending forward about two inches into the frontal bone, accompanied with perfect coma. Pupils dilated, the left more than the right ; and insensible to light, with hemiplegia of the right side ; pulse 40 per minute and barely perceptible. I immediately ordered hot applications to the body and extremities, consisting of sinapisms, friction, and hot blankets.

As there was but slight depression, and no comminution of the bone discoverable near the seat of injury, I thought it best to defer any operative procedure until something more definite could be made out. For this purpose Dr. Heise was summoned to meet me in consultation. Meantime, so soon as reaction had become somewhat established, I bled the patient about twenty ounces ; which produced slight amelioration of the symptoms, so much so as to admit of fluids being swallowed. Upon the arrival of Dr. Heise, he advised a second bleeding, which had the effect of increasing the circulation from 40 to 58 pulsations per minute. Otherwise to keep the bowels open, the urine frequently drawn, and trust to nature.

From this the patient remained in about the same condition for four weeks, when consciousness began to return, and in six months from the time of injury he had so far regained his mental faculties as to be able to attend to most of his business, and the hemiplegia had entirely passed off.

*Case* 6.—Feb. 14th, 1868, Mr. C., a short, thick-set, muscular Irishman, walked into my office upon crutches, and gave me the following history : Two and a half months previous, while at work in the "B" coal shaft, a large stone from the roof fell upon his back and hips, crushing him to the earth. He was immediately discovered and taken to his boarding house. His friends, thinking that he was not seriously injured, neglected to call a physician, and up to the present time he had not been examined by any one competent to form a diagnosis.

A careful survey of the case revealed a dislocation of the head of the left femur upon the dorsum of the ilium. I made arrangements to have him call the next morning, when, with the assistance of Drs. Johnson and Fox, we would reduce the luxation. After leaving my office, his friends advised him to go to Chicago. This advice he immediately followed. While in Chicago, reduction by manipulation was thoroughly tried, after which "Jarvis's Adjuster" was applied, and the extension continued until, as the patient expressed it, he "thought they would pull his leg off," and all to no purpose. He remained in Chicago under treatment some ten or twelve days, and then returned home as he went, positively assured by those under whose treatment he had been, that the dislocation could not be reduced. From this I lost sight of the patient for some three and a half months, making six months from the time of accident.

Meantime I had talked with the late Dr. Johnson about the case, and had arranged with him to assist in an attempt at reduction, provided we could secure the patient's consent. One morning, when near the doctor's office, we met the man—with his crutches, as usual—and induced him to walk in; when, after a careful examination of the limb, and much persuasion, we obtained his consent to give us a trial.

Drs. Fox and Campbell were called in consultation—the man placed upon his back on a lounge, and brought fully under the influence of chloroform; the leg grasped under the knee and flexed upon the abdomen; the knee bent to a right angle, whereby the leg below the knee was used as a lever, which, being inclined outward by a gentle pressure, caused the head of the femur to slide around under the acetabulum, and drop into that cavity with an audible snap: the whole accomplished in a very short space of time. After reduction, the patient was placed under the charge of Dr. Campbell, who was attending the miners at the time. From this the patient rapidly regained the use of his leg.

*Case* 7.—On the 19th of August, 1869, during the horse fair at this place, A. F., one of the riders, fell from his horse, and was struck on the head by the hoof of a passing horse. He was immediately picked up in an insensible condition, with eyes turned up, and spasmodic twitching of all the muscles. I saw the boy in twenty minutes from the accident, in connection with Dr. Lecaron. At this time he was struggling in a violent convulsion. Sinapisms were freely applied to the back and limbs, in connection with warm blankets—and cold to the head—all to no purpose, as the convulsions continued at intervals of from five to ten minutes, and lasted from ten to twenty minutes each time. Upon a careful examination of the head, we discovered a fracture of the left temporal bone, directly over the ear, with free oozing of blood from both ears. No depression or comminution of bone was discovered through the scalp wound some three inches in length over the seat of fracture.

After some time spent in fruitless efforts to relieve the spasms, we bled the patient freely. This only had the effect to slightly lengthen the interval between the paroxysms. Pulse at this time 50 per minute and almost imperceptible. Pupils slightly contracted.

So far, everything tried having failed to relieve the urgent symptoms, we administered ether until full anæsthetic effect was produced. This controlled the paroxysms at once. Finding the effect so perfect, we continued the inhalation for several days, sufficient to keep the patient partially under its influence, and until all muscular twitchings had ceased. Meantime the water was drawn as occasion required, and the bowels relieved by injections. After six days of more or less constant etherization, consciousness began to return. From this time the patient rapidly improved, and in four weeks he was up and so far recovered as to require no further treatment.

*Case* 8.—A. E., a small, muscular man, of sanguine temperament, while riding home upon the evening of June 30th, 1871, accidentally fell from the top of a double wagon box—about eight feet in height—to the ground, striking upon the back of his head and neck. He was immediately picked up, when it was found that he could not walk, and the bystanders supposed that he was intoxicated, as he could converse as well as usual. He was conveyed to a neighboring house, where I saw him a short time after the accident, lying upon a mattress, with eyes and face natural; pulse normal. Upon extending the examination further, I discovered that all the muscles of the limbs and trunk were paralyzed; that sensation was also gone below the shoulders, and that respiration was entirely performed by the diaphragm. He complained of severe pain in the region of the fifth cervical vertebra, whenever the head was moved, or pressure made at that point.

On the fifth day sensation had slightly returned in the right forearm; on the following day he complained of his right arm being "asleep," and could move the index finger; on the seventh day the abdomen became tympanitic, and the right leg had the same pricking sensation as was felt in the right arm on the fifth day; on the fifteenth day he could move the toes of the right foot, and all the fingers of the right hand. Twenty days from the accident he was able to micturate without assistance. In three months he could move his right arm and leg quite well, and the left leg sufficient to take one or two steps by supporting himself. The tympanites had become entirely relieved, and the bowels moved regular every day without injections.

Treatment consisted in cupping the spine every second or third day, for the first week or two, followed with electricity and strychnia after a few weeks. Meantime the bowels were relieved by injections, and the water drawn whenever (during the early part of the treatment) it was found necessary.

*Remarks.*—The question naturally arose in cases five and seven, whether the base of the skull was fractured or not. The prevailing opinion seemed to be that it was, on account of the hemorrhage from the ears, nose, and mouth, together with the other grave symptoms, although the very common symptom of bleeding from the ears is well known not to be a constant one in these cases. Another very important question presented itself in the above named cases, as to whether they would be benefited by trephining. When the extent of injury, and alarming symptoms, are taken into consideration, one would be tempted to operate, although no depression of bone could be discovered, could it be ascertained for a certainty that there was no fracture of the base of the skull, notwithstanding fractures in that region have been proven not to be so unavoidably and uniformly fatal as was formerly supposed to be the case.

